# Research progress on the function of non-muscle myosin II during cell division

**DOI:** 10.3389/fcell.2026.1852038

**Published:** 2026-08-20

**Authors:** Jingjing Ding

**Affiliations:** Institute of Ecology and Health, Hangzhou Polytechnic University, Hangzhou, Zhejiang, China

**Keywords:** aneuploidy, cell rounding, cytokinesis, non-muscle myosin II, tumorigenesis

## Abstract

Myosin is a superfamily of motor proteins that has attracted extensive research interest for many years due to its ability to efficiently convert chemical energy into mechanical energy during muscle contraction and various other movements in eukaryotic cells. Non-muscle myosin II (NMII) consists of three members, NMIIA, NMIIB, and NMIIC, which are positioned at the downstream end of numerous signaling pathways and plays a central role in cell adhesion, migration, and division. During cell division, NMII serves as the primary protein that generates mechanical force. It participates not only in cell rounding from prophase to metaphase but also interacts with actin to assemble a contractile ring during late stages, which then contracts to split the mother cell into two daughter cells. Loss of NMII function may result in cell division failure and consequently tetraploidy. Subsequent division of tetraploid cells can generate aneuploidy, which is closely associated with tumorigenesis as shown in many studies. In this review, we will discuss the roles of NMII throughout the entire cell division cycle.

## 1 Introduction

Myosin, a member of the motor protein superfamily, plays an important role in the processes that require force production in cells or tissues, such as cell division, migration and adhesion. Myosin slides along filamentous actin (F-actin) and generates tension in an energy-consuming process, with the required energy supplied by the hydrolysis of adenosine triphosphate (ATP) via the intrinsic ATPase activity of myosin (Arakelian et al., 2015; Richards and Cavalier-Smith, 2005). In addition, myosin can indirectly interact with actin to recruit cell adhesion-related proteins like integrins and other signaling molecules to adjacent sites, thereby exerting synergistic effects (Kutzner et al., 2024; Lewis et al., 2024; Tokuo, 2020).

Some members of the myosin superfamily belong to class II myosin, which, together with actin, forms the main contractile units of cardiac, skeletal, and smooth muscle. Sliding cross-bridges connect thicker myosin filaments to thinner actin filaments to power physiological processes such as blood pumping. Moreover, the ability to form myosin filaments is a unique feature of class II myosin that distinguishes it from other myosin classes. In non-muscle eukaryotic cells, myosin II is also present and is structurally and functionally similar to its muscle counterpart. However, non-muscle myosin II forms thinner filaments than muscle myosin II and consequently generates less force (Kokate et al., 2022; Niederman and Pollard, 1975; Pasapera et al., 2022). Like muscle myosin II, non-muscle myosin II (NMII) is composed of three pairs of peptide chains: two heavy chains (230 kDa), two regulatory light chains (RLCs, 20 kDa) that regulate NMII activity, and two essential light chains (ELCs, 17 kDa) that stabilize the heavy chain structure (Sellers and Heissler, 2019). Although these myosins are referred to as “non-muscle” myosin to distinguish them from myosin derived from muscle cells, NMII is also present in muscle cells and play different roles at different stages of skeletal muscle development and differentiation. However, the function of NMII may be abnormal for some unknown reasons, leading to the occurrence and development of a variety of diseases, such as deafness, cataract, cancer and a variety of inflammatory conditions. In addition, some pathogens, such as HIV, hepatitis C virus, dengue virus, lymphoma virus and *Salmonella*, can also cause disease by controlling NMII (Betapudi, 2014; Feroz et al., 2024).

In addition to its involvement in cell migration and cell adhesion, NMII is also involved in the whole cell division process due to its unique functions. During division, cells transition from a flat morphology in the G2 phase to a spherical shape in metaphase, before ultimately being divided by the contractile ring. Each of these morphological changes requires substantial mechanical force, primarily generated by the interaction between NMII and actin (Pollard and O'Shaughnessy, 2019). Cell division is a finely regulated event that must be executed with high fidelity to ensure genome stability. After the mitotic apparatus divides the replicated chromosome into independent nuclei, the contractile ring on the cell division furrow distributes the daughter nuclei between the two daughter cells (D'Avino et al., 2015). Accurate and equal allocation of daughter nuclei to both daughter cells is required for normal cell survival and avoidance of aneuploidy events. Pioneering experiments over 4 decades ago showed that dividing mammalian cells assemble a contractile ring consisting of actin filaments and NMII near the equatorial plate and that these two proteins are indispensable for the contractile ring (Schroeder, 1973; Sellers and Heissler, 2019). Mechanical measurements reveal that the contractile ring in echinoderm eggs generates a contractile force of approximately 25 nN. Although significantly smaller than the force produced by muscle cells, this tension is fully sufficient to cleave the dividing cell into two daughter cells (Rappaport, 1967). Therefore, the whole process of cell division depends on the participation of NMII, and the abnormal function of NMII will lead to the failure of cell division. When mammalian cells enter mitosis, they first round from a flat state to a spherical shape in morphology. This morphological change is often accompanied by changes in the mechanical properties of the cell. Studies using different measurements have shown a significant increase in cell surface tension, hydrostatic pressure, and cell cortex stiffness upon entry into mitosis (Liu et al., 2019; Stewart et al., 2019).

In summary, NMII participates in multiple stages of cell division, ranging from the initial rounding of the cell at the onset of division to the final cytokinesis that produces two genetically identical daughter cells. Dysregulation or abnormal function of NMII at any of these stages may lead to failed cell division and the generation of tetraploid cells. The subsequent division of these tetraploid cells can further give rise to aneuploid cells, which are closely associated with tumor initiation and progression. Therefore, this review will summarize the roles of NMII throughout different stages of cell division and discuss the pathological consequences resulting from its dysfunction.

## 2 Structure and function of myosin

**FIGURE 1:**
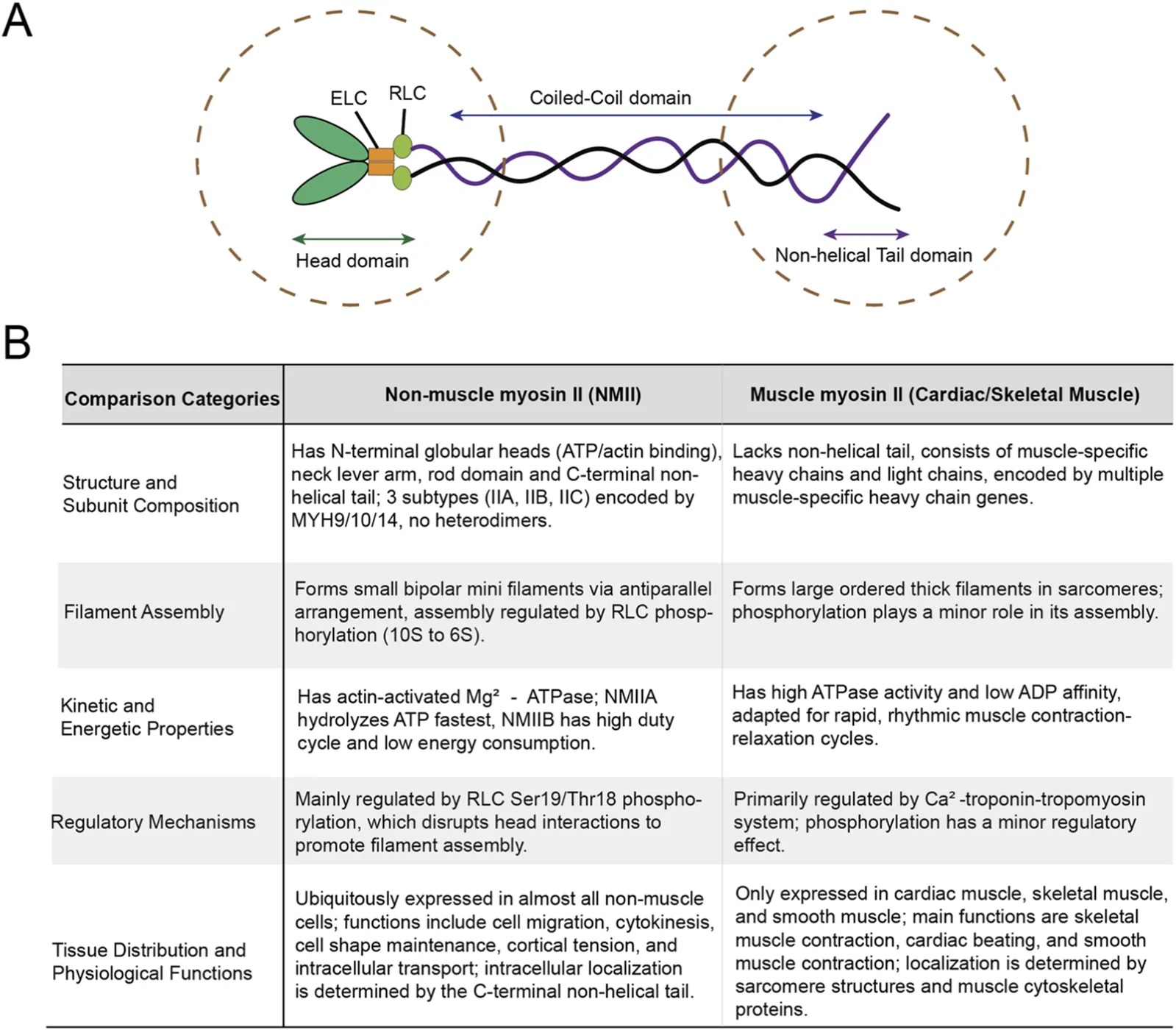
**(A)** Schematic diagram of the structural characteristics of myosin II. **(B)** Comparative table summarizing the differences in structure, function, and diseases caused by functional abnormalities between muscle myosin II and non-muscle myosin II.

The special structure of NMII is closely related to its complex function, in which two globular head domains contain binding sites for ATP and actin, and each domain is capable of binding two light chains with different functions. When chemical energy from ATP is converted into mechanical energy to move the NMII head, its neck region acts as a lever arm to amplify the rotational motion of the head. The NMII neck domain is followed by a long α-helix coil, which is coiled to form an extended rod domain that affects the dimerization of the heavy chain and the non-helical tail. This rod-like domain can bind by antiparallel arrangement and form bipolar NMII mini filaments that are much smaller than those found in cardiac and skeletal muscle cells (Wang et al., 2020) (Figure 1).

Recent high-resolution imaging studies have revealed that the higher-order organization of NMII filaments is highly dependent on the cell type and physiological context. Unlike the uniform thick filaments in muscle cells, NMII in adherent fibroblasts often assembles into ordered, sarcomere-like stacks to generate strong traction forces (Dasbiswas et al., 2018; Fenix et al., 2016). In contrast, during cytokinesis in early embryos (such as *C. elegans*) and epithelial cells, NMII and F-actin form dynamic, discontinuous cortical nodes that condense into a continuous contractile ring via a search-and-capture mechanism (Taneja et al., 2020). These distinct organizational modes highlight the structural plasticity of NMII, allowing it to adapt to the specific mechanical requirements of different cell types.

There are three different myosin heavy chain genes (myosin heavy chain 9, MYH9, MYH10 and MYH14) in mammalian cells that encode NMII heavy chain proteins: Non-muscle myosin heavy chain IIA (NMHCIIA), NMHCIIB and NMHCIIC, respectively. However, in some species, such as *Drosophila melanogaster*, there is only one NMHCII gene, zipper17 (Mansfield et al., 1996). We refer to the entire non-muscle myosin II molecule (including the heavy chain and light chain) as NMII, and the heavy chain itself as NMHC (non-muscle myosin heavy chain). The NMHC isoforms determine the NMII isoforms, named NMIIA, NMIIB, or NMIIC accordingly, in which the light chain and heavy chain must bind to each other to maintain the normal native active conformation of NMII. Specific NMHCII deletions will result in NMII isoform defects (Jordan and Lin Shao, 2014).

In mammalian cells, the different isoforms of NMHCII differ greatly in the shorter, nonhelical tail regions, so that these sequences can be mimicked by synthetic peptides to generate subtype-specific antibodies. At present, there is no evidence that heterodimers can be formed among the three NMHCII subtypes, and immunoprecipitation experiments with subtype-specific antibodies on the subtype mixture can only detect antigen-specific subtypes (Golomb et al., 2004). NMII has two important kinetic properties, namely, Mg^2+^ ATPase activity activated by actin (increased ATP hydrolysis when myosin binds to actin) and duty cycle (the time that myosin and actin are in a bound state, when both are in a bound state, they can generate mechanical force) (Yamamoto et al., 2019). Of the three NMII isoforms, NMIIA has the highest rate of ATP hydrolysis and drives actin filament sliding more rapidly relative to NMIIB and NMIIC. The duty cycle of NMIIB is significantly higher than that of NMIIA and NMIIB has an extremely high affinity for ADP compared to other myosins, because ADP release by NMIIB is significantly slowed under mechanical load. Specifically, this occurs when actin filaments exert backward tension, pulling the myosin head in the opposite direction of its power stroke. Therefore, NMIIB is more suitable than NMIIA for long-term force production on actin filaments and consumes less energy (Zhang et al., 2017). Both the ATPase active center and the motor active center of NMII are located in the N-terminal domain, whereas the nonhelical tail of the C-terminal rod region determines myosin filament assembly and the intracellular localization of the NMII isoform. Chimeric experiments of NMIIA and NMIIB show that the intracellular localization of NMII isoforms is determined by amino acids 179 to 190 in the C terminus, and this peptide chain contains a rod-like assembly active domain and a non-helical tail region (Sandquist et al., 2008).

**FIGURE 2:**
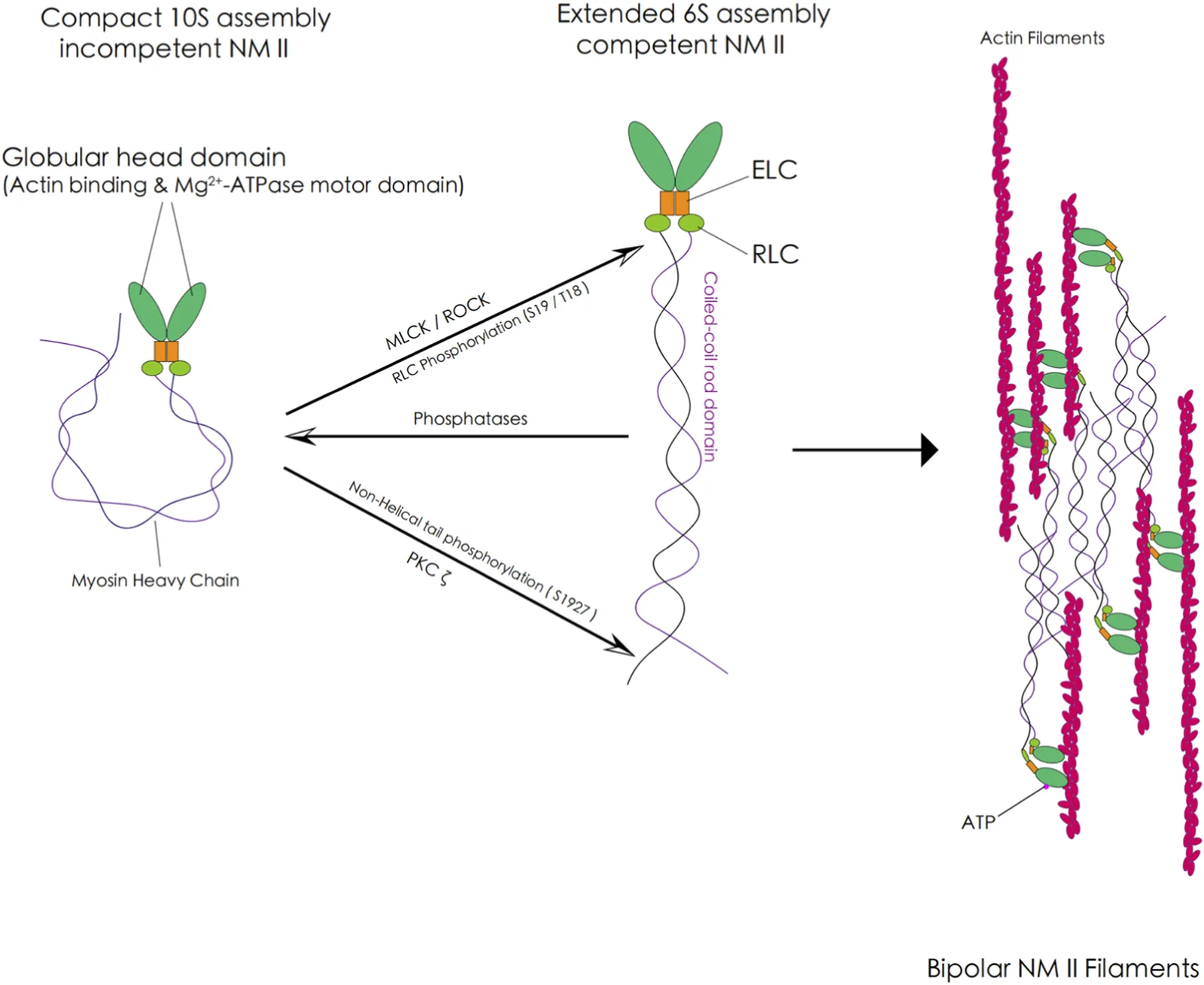
NMII interacts with Actin after conformational transformation. In the presence of ATP, the unphosphorylated NMII folds into a tightly compressed conformation (incompetent NMII), where one head locks onto the other through asymmetric interactions, and its nonhelical tail also folds at these two points and interacts with the head to further compress protein molecules to impede myosin filament assembly. RLC phosphorylation disrupts head-head and head-tail interactions, turning the folded NMII (10S) into an elongated competent NMII (6S), resulting in the formation of NMII bipolar filaments in ionic strength under physiological conditions.

Regulation of NMIIA, NMIIB, and NMIIC depends on reversible phosphorylation of the regulatory light chain (RLC) Ser19. Phosphorylation of this site significantly increased the Mg^2+^-ATPase activity of NMII in the presence of actin by altering its head conformation, but had little effect on the affinity between NMII and actin. Thr18 of NMII can also be phosphorylated, and double-site phosphorylation of Ser19 and Thr18 of RLC is commonly seen in cultured cells in the laboratory. On the premise of Ser19 phosphorylation, Thr18 phosphorylation can further increase the actin-activated Mg^2+^-ATPase activity (Nava et al., 2020). *In vitro* studies have also shown that RLC phosphorylation regulates the assembly of NMII mini filaments. In the presence of ATP, the unphosphorylated NMII folds into a tightly compressed conformation, where one head locks onto the other through asymmetric interactions, and its nonhelical tail also folds at these two points and interacts with the head to further compress protein molecules to impede myosin filament assembly. RLC phosphorylation disrupts head-head and head-tail interactions, turning the folded, rapidly precipitated molecule (10 S) into an elongated, slowly precipitated molecule (6 S), resulting in the formation of NMII bipolar filaments under physiological ionic strength. Bipolar filaments consisting of 14–20 NMII molecules facilitate the interaction of NMII filaments with actin filaments, thereby forming the basic unit of force production to promote mechanical force generation (Vicente-Manzanares et al., 2009) (Figure 2).

So far, more than ten kinases that can phosphorylate NMII RLC have been reported, including MLCK (Myosin light chain kinase, MLCK), ROCK (Rho-associated, coiled coil containing kinase, ROCK) and citron kinase (CIT-K), etc. (Zhang et al., 2017). These kinases phosphorylate either Ser19 or Thr18 or both sites to release the inhibition of NMII by unphosphorylated RLC. Protein kinase C (PKC) can also phosphorylate RLC, which is different from the above kinases in that it can phosphorylate Ser1, Ser2 and Thr9 sites of RLC, which makes RLC a non-ideal phosphorylation substrate for MLCK to reduce NMII activity and thus promote actomyosin filament recombination (Beach et al., 2011). In addition to RLC, PKC phosphorylates NMII heavy chains, although NMII heavy chain (NMHCII) phosphorylation plays an important role in regulating NMII activity, the non-helical tail of NMHC, is the phosphorylation site of CKII, and phosphorylation of this amino acid residue inhibits the assembly of NMIIA rod regions into filaments (Javier-Torrent et al., 2019).

PKCζ has been shown to phosphorylate NMIIB under the regulation of p21-activated kinase 1 (PAK1) in prostate cancer cell lines. Phosphorylation at the Ser1935 residue in the non-helical tail region of NMHCIIB is also inhibited by epidermal growth factor (EGF), and this phosphorylation slows down the assembly of myosin filaments in cells (Theos et al., 2005). Thus, many regulatory events of NMII depend on the phosphorylation of its heavy chain, particularly in the non-helical tail region (Figure 2). Collectively, precise phosphorylation of both the heavy and light chains of NMII is essential for its proper function.

Despite these well-characterized biochemical differences, a major unresolved paradox in the field is the degree of functional redundancy among NMII isoforms. While *in vitro* data strongly suggest strictly divergent roles based on distinct motor kinetics, *in vivo* genetic depletion models often show that overexpressing NMIIA can partially rescue NMIIB deficiency, and *vice versa*. We argue that the current evidence points toward a “threshold model” of contractility rather than absolute isoform specificity, and the cell requires a critical total level of NMII cross-linking and motor activity, with the specific ratio of IIA/IIB fine-tuning the mechanical output rather than acting as strict binary switches. Furthermore, while the activating role of RLC phosphorylation is universally accepted, the physiological significance of heavy chain phosphorylation remains a weak point in current literature, lacking robust *in vivo* validation and representing a critical gap in our understanding of NMII spatiotemporal dynamics (Heissler and Sellers, 2016).

## 3 Myosin participates in the process of cell rounding

**FIGURE 3:**
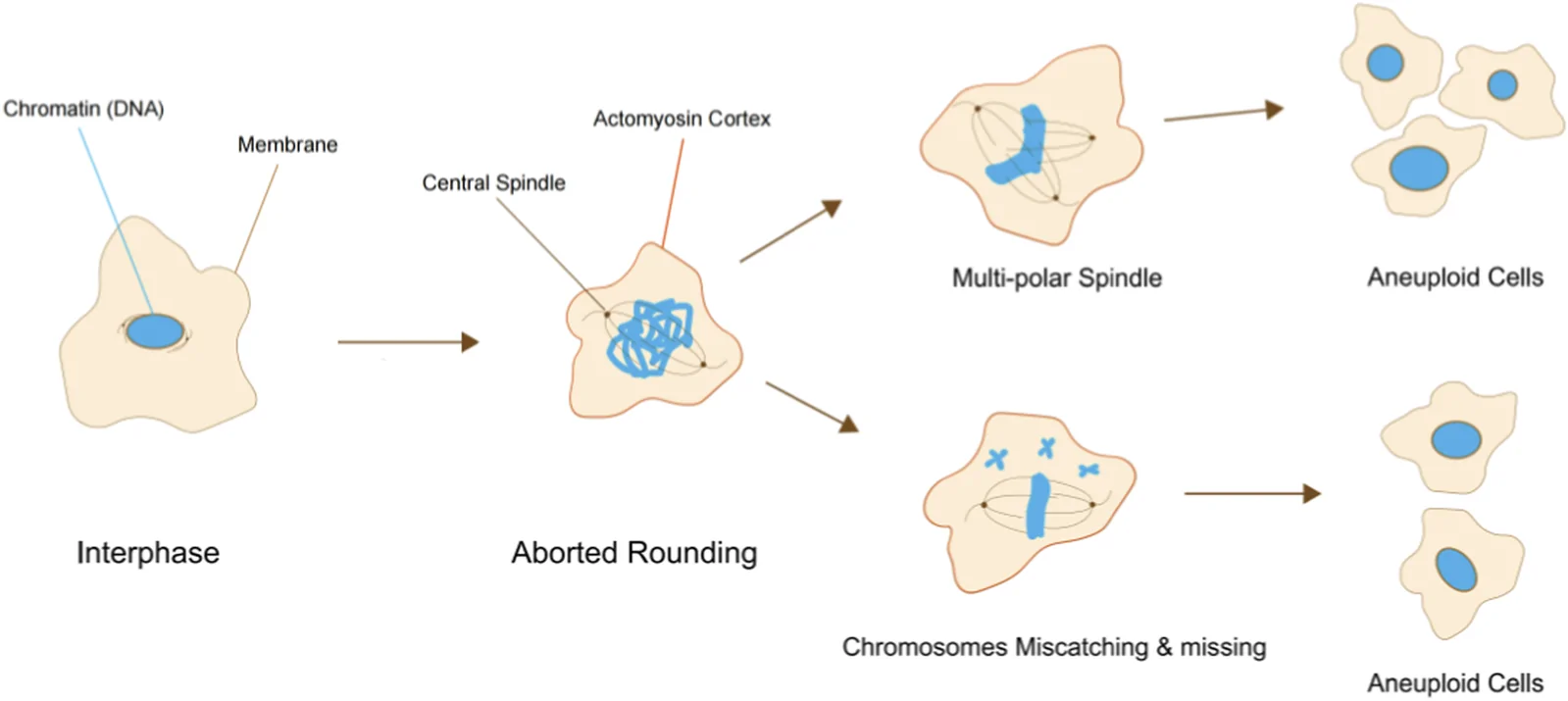
Effect of cell rounding defects on spindle formation and cell division. Cell division is accompanied by a series of drastic morphological changes, and the first step of cells entering mitotic prophase is to round into a spherical shape in morphology. The failure of cell rounding will lead to the abnormal division process and the generation of aneuploid daughter cells due to the insufficient space for assembly of the spindle.

The previous section discussed the structure and function of myosin, which interacts with actin as the main force-generating unit of the cell and is involved in various cellular activities, including cell division. The process of cell division is accompanied by a series of drastic morphological changes. For example, the first step as cells enter mitotic prophase is to round into a spherical shape; failure of cell rounding leads to an abnormal division process and the generation of aneuploid daughter cells due to insufficient space for spindle assembly (Figure 3). As adherent cells enter mitosis, focal adhesion complexes containing canonical integrins that anchor them to the extracellular matrix are disassembled, after which cell rounding begins. Dividing cells remain loosely connected to contractile fibers containing β1-integrin (Dix et al., 2018). However, cell rounding is not merely a consequence of reduced adhesion forces, many studies have demonstrated that the process also involves changes in cell surface tension. Accordingly, a variety of techniques have been employed to measure surface tension fluctuations during cell rounding (Taubenberger et al., 2020).

In recent decades, various techniques have been utilized to characterize cell mechanics. While atomic force microscopy (AFM) is widely used for flat, adherent cells, it has significant limitations for spherical or weakly adherent mitotic cells (Cartagena-Rivera et al., 2016; Muller et al., 2021). For these cell types, approaches such as micropipette aspiration (MPA) are necessary. For instance, classic studies have elegantly employed MPA to demonstrate how interactions between myosin II and actin crosslinkers control contractility dynamics and mechanics during cytokinesis (Evans and Robinson, 2018; Reichl et al., 2008). With the support of the above techniques for measuring cell surface tension, research on how cell surface tension is generated has increased year by year. Cell surface tension contains cell cortical tension, which is the major component of cell surface tension, and the tension generated by the plasma membrane, which is much smaller than the cell cortical tension. Strictly defined, cortical tension represents the thermodynamic energy cost of adding a unit of surface area to the cell. This tension is primarily generated by the actomyosin cortex, a dynamic network of filamentous units attached to the inner leaflet of the plasma membrane (Kelkar et al., 2020). The depolymerization of actin filaments eliminates the mechanical differences between interphase and mitotic cells, making the cells extremely pliable (Chugh et al., 2017). Furthermore, it is important to note that the mitotic spindle and the cortex are dynamically linked, and disrupting microtubules directly impacts cortical mechanics. Specifically, microtubule signaling interconnects with the myosin II network to coordinate mechanical properties, demonstrating that spindle integrity is a crucial upstream modulator of cortical tension during cytokinesis (Zhou et al., 2010). This suggests that the actomyosin cortical network plays an important role in controlling cell shape, mechanical force properties, and the ability to resist deformation (Fischer-Friedrich et al., 2016), but how is this coordinated at the molecular level? The transition to a rigid, rounded mitotic cortex is tightly orchestrated by the RhoA-ROCK signaling axis. Upon mitotic entry, the guanine nucleotide exchange factor Ect2 activates RhoA globally at the cell cortex (Matthews et al., 2012). This active RhoA-GTP engages downstream effectors, notably Rho-associated kinase (ROCK). ROCK subsequently phosphorylates the regulatory light chain (RLC) of NMII while concomitantly inactivating myosin phosphatase (Maddox and Burridge, 2003). This specific signaling cascade triggers massive actomyosin network reorganization, converting the polarized interphase cell into a uniformly highly contractile spherical structure before the contractile ring is even assembled.

Recent studies have shown that the differences in structure, contractile properties and protein composition of the cell cortex may be the main reasons for the differences in biophysical properties between interphase and mitotic cells. In mitosis, the cortical layer of the cell is thinner than that in interphase, but it can produce greater cortical tension (Chugh et al., 2017). A key determinant of cortical tone, as found by interfering with actin regulators, is actin filament length. Shortening the length of actin filaments, such as knocking down the nucleating protein Diaph1, leads to cortical thinning and reduced cortical tone (Rosa et al., 2015). Interestingly, lengthening actin filaments to thicken the cellular cortical layer by knocking out the actin cap-forming protein CAPZB or Cofilin also resulted in a decrease in cortical tone, suggesting that the changes in mechanical properties observed in mitosis due to actin filament length are not caused by simply shortening or lengthening the filaments. Rather, it is achieved by regulating the balance between actin filament assembly, depolymerization, branching, severing, and capping (Chugh et al., 2017). Proteomics studies have shown that the cell cortex contains many regulatory factors related to actin filament assembly, cutting and capping (Serres et al., 2020). High-throughput screening experiments have shown that many of these regulators are indispensable for cortical tension generated by mitosis (Koenderink and Paluch, 2018). In addition, cross-linking between proteins is also critical for the generation of cortical tension. Computational simulations of the cortical layer structure show that cortical network connectivity is key for the generation of higher cortical tension. Moreover, actin filament cross-linking proteins, such as Fascin and a-Actinin, are key factors for the generation of higher cortical tension. Knocking down them can significantly reduce the cortical tension of mitotic cells (Chugh et al., 2017).

One of the main functions of actin filaments in the cellular cortex is to support myosin contraction. Myosin filaments within the actomyosin network can not only participate in cortical network cross-linking, but also act as motor proteins to allow microfilaments to slide against each other (Svitkina, 2020). In the process of mitotic cell rounding, NMII gradually accumulates in the cortical layer of cells, and the intracellular pressure also increases (Ramanathan et al., 2015). Therefore, the accumulation of NMII in the cortical layer is crucial for the generation of cortical tension, and the knockdown of NMII can reduce the cortical tension of cells by more than 90% (Chugh et al., 2017). Our results showed that treatment of dividing cells with Blebbistatin, a specific NMII inhibitor, significantly decreased both the accumulation of NMII in the cortical layer and the resulting surface tension that maintains cell spherical morphology. Analysis of rheological characteristics showed that after Blebbistatin treatment, the cortical characteristics of dividing cells were more solid, but the hardness was very low (Fischer-Friedrich et al., 2016). Conversely, the RhoA activator Calpeptin increases intracellular pressure and cortical contractility. This heightened contractility and resulting cortical instability subsequently induce the formation of blebs on the surface of mitotic cells (Ramanathan et al., 2015; Shah et al., 2024). During mitosis, NMII increases cellular cortical tone by both promoting cross-linking of the cortical network and applying contractile forces to the network, and these two functions are not easily separated because cross-linking of the actomyosin network also affects NMII contractility (Koenderink and Paluch, 2018).

Historically, two somewhat conflicting models have been proposed to explain the generation of mitotic cortical tension: the “motor-driven” model, emphasizing the ATPase-dependent sliding of actin filaments by NMII, and the “cross-linking” model, which posits that NMII acts primarily as a passive structural tether (Charras and Yap, 2018). Based on recent high-resolution rheological evidence, we propose an integrated view: neither model acts in isolation. During early prophase, the rapid increase in tension is predominantly motor-driven, heavily reliant on NMII ATPase activity to compact the network. However, once the spherical geometry is achieved, the cortex transitions into a dynamically cross-linked steady state where the high duty-cycle of NMII (particularly NMIIB) maintains tension with minimal ATP consumption. This synthesis reconciles the seemingly contradictory observations that inhibiting motor activity blocks initial rounding, while long-term maintenance of the rounded state is more sensitive to cross-linking disruptions.

## 4 Non-muscle myosin II participates in the cytokinesis process

The previous section discussed that myosin interacts with actin and forms the actomyosin cortical network under the plasma membrane, that is the cell cortical layer. The contraction of the cortical layer generates cortical tension, which makes the cell gradually round from the flat state of interphase to the spherical state of prometaphase (Taubenberger et al., 2020). The cytokinesis process is the final step of the cell cycle in which the cellular genome, organelles, and plasma membrane are allocated to the two daughter cells. This is not only indispensable for the maintenance of ploidy during metazoan development and adulthood, but also essential for the avoidance of disease states, including cancer (Bielski et al., 2018). When the cell enters the anaphase of division, the division plane is first identified, which is determined by the spindle and the activated form of RhoA (the GTP-binding form of RhoA) targeting the equatorial plate plasma membrane (Gomez-Cavazos et al., 2020). Activated RhoA (RhoA-GTP) recruits Formin and Anillin to the division site and simultaneously activates kinases of NMII, such as ROCK. Therefore, the entire cleavage furrow location process depends on the accurate localization of activated RhoA, which is regulated by signals from microtubule structures (the spindle and stellate microtubules at anaphase). The relative contribution of these two microtubule populations is highly cell-type dependent; larger cells (such as embryonic blastomeres) rely primarily on astral microtubules, whereas smaller somatic cells depend more on signals from the central spindle (Horvath et al., 2017; Manukyan et al., 2018; Skrzypiec-Spring et al., 2024; Wang et al., 2023). Anaphase spindle signals originate in the central spindle midzone, a narrow region where antiparallel microtubule ends (plus ends) overlap. It recruits Centralspindlin and CPC (chromosomal passenger complex) to the region. CPC is one of the central regulators in mitosis, including Aurora B core kinase, scaffolding protein INCENP and two targeted proteins, Borealin and Survivin. CPC has different localizations and functions in different mitotic stages. At anaphase, CPC localizes to the spindle midzone to promote chromosome segregation, while at telophase, CPC participates in the formation and abscission of the midbody (Basant et al., 2015; Goto et al., 2003). When MKLP1 S710 is phosphorylated in the Centralspindlin complex (a heterotetramer of MKLP1 and RACGAP1) in dividing cells, 14-3–3 inhibits Centralspindlin complex formation by binding to MKLP1 and interferes with cytokinesis. Aurora B kinase in CPC deregulates the inhibitory binding of 14-3-3 by phosphorylating MKLP1 at S708. Thus, the downstream pathways are activated to promote cytokinesis (Basant and Glotzer, 2018).

First, the two complexes promote the Rho Guanine nucleotide exchange factor (GEF) ECT2 interacts with RACGAP1 (Rac GTPase-activating protein 1, RACGAP1) subunits in the Centralspindlin complex. It recruits ECT2 to the adjacent plasma membrane and activates RhoA located near the plasma membrane (Kumar et al., 2015; Mishima, 2016). At present, the molecular mechanism of how ECT2 reaches the plasma membrane of the equatorial plate from the spindle at anaphase is not clear, but it may assist diffusion or active transport along actin filaments (von Dassow et al., 2009). Local activation of RhoA by ECT2 counteracts the overall inactivation of RhoA by RhoGAP and ensures that activated RhoA is only localized in the equatorial region, as RhoA diffusing from the equatorial plate is inactivated again by encountering widely distributed Rhogaps (Green et al., 2012; Miller and Bement, 2009).

Activated RhoA-GTP binds to the plasma membrane through modification of its lipid C-terminus and activates effector proteins. For example, RhoA binds and activates Formin, which promotes actin nucleation and polymerization, as well as Rho-associated kinases and CIT-K. These kinases phosphorylate the regulatory light chain (RLC) of NMII, and Rho kinases further phosphorylate myosin light chain by phosphorylating phosphatase that inhibits myosin phosphorylation. Actin filaments (F-actin) and myosin (which form a functional unit called actomyosin) interact to form a mitotic contractile ring, a stage-specific, temporary organelle associated with the plasma membrane (Duan et al., 2016; Lacroix and Maddox, 2012). Actomyosin can generate contractile force and promote contraction of the contractile ring, like that of sliding filaments in muscle. The progressive contraction of the contractile ring will gradually contract the plasma membrane and eventually close the cytoplasmic junction between the two daughter cells. Although NMIIA and NMIIB can co-assemble into heterotypic filaments, they exhibit a distinct division of labor during cytokinesis due to their different kinetic properties. Recent studies in human cell lines demonstrate that NMIIA, with its high ATPase activity, is rapidly recruited to the equatorial cortex to drive the initial, fast ingression of the cleavage furrow (Yamamoto et al., 2019). Conversely, NMIIB is recruited later to the furrow. Owing to its high duty cycle and low ADP release rate, NMIIB acts as a tension sensor and cross-linker, generating sustained contractile force to maintain the furrow architecture and stabilize the intercellular bridge during the slow maturation and abscission phases (Taneja et al., 2020; Wang et al., 2019). This spatiotemporal coordination ensures both the efficiency and structural integrity of the cytokinesis process. The role of NMIIC remains less characterized, though it may assist in the final abscission steps.

**FIGURE 4:**
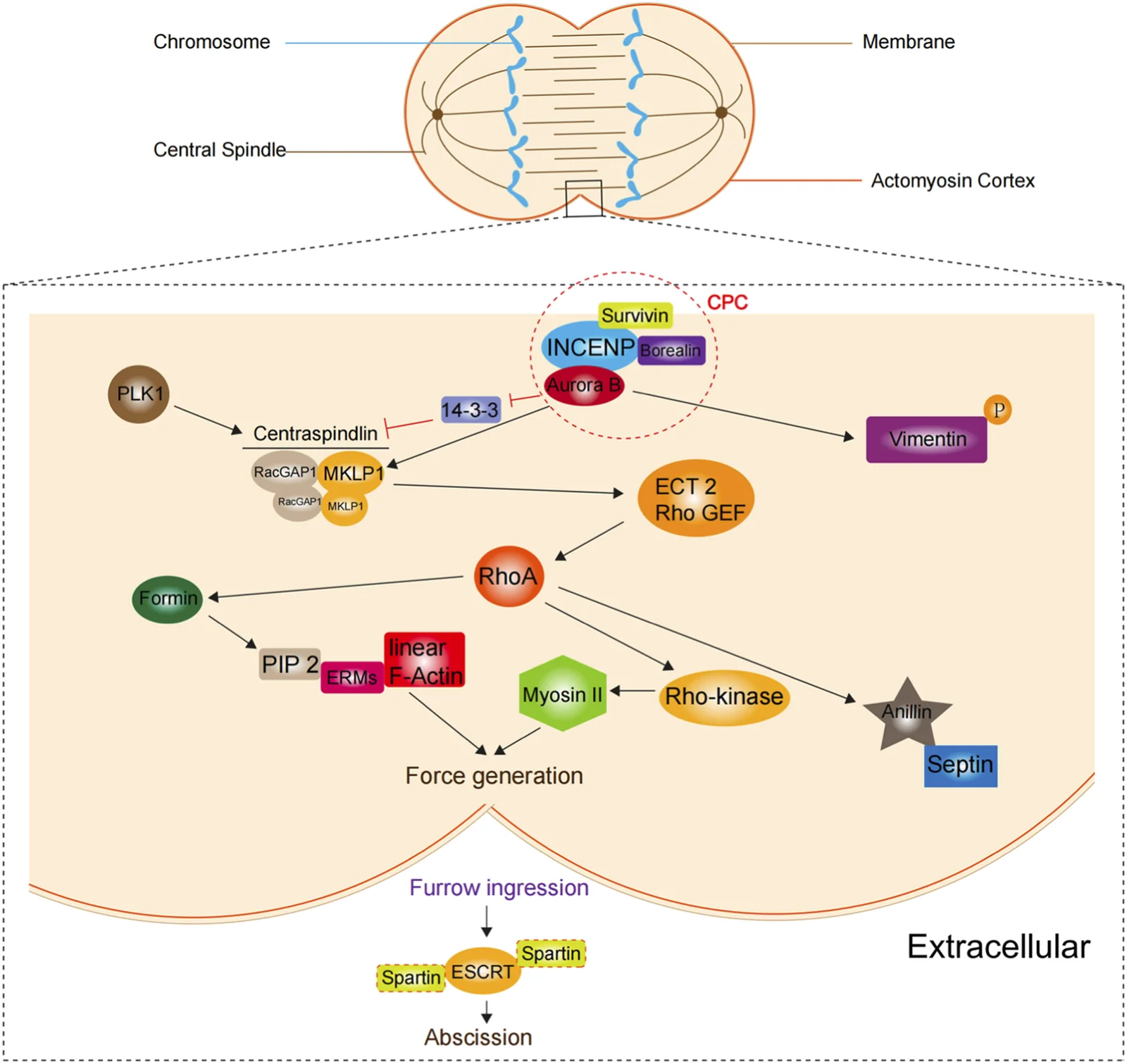
Important associated proteins and protein complex involved in cleavage furrow hanging and ingression during cytokinesis.

While NMII serves as the primary force generator, its spatial organization and effective force transmission to the plasma membrane strictly depend on an integrated scaffold network, predominantly orchestrated by Anillin and Septins. Anillin functions as a central cross-linker that simultaneously binds RhoA, F-actin, NMII, and Septins, effectively coupling the actomyosin contractile machinery to the equatorial membrane (Kechad et al., 2012; Piekny and Maddox, 2010). Septins, forming membrane-bound diffusion barriers and non-polar filaments, work synergistically with Anillin to restrict the localization of contractile elements and adapt to the negative membrane curvature during furrow ingression (Addi et al., 2018; Pollard and O'Shaughnessy, 2019). Thus, rather than being peripheral actors, the Rho-Anillin-Septin network forms a vital functional module; without this scaffold, NMII-driven contraction would lead to ring instability and cytokinesis failure. Moesin and other ERM proteins also contain sites for binding phosphoinositides and F-actin and may therefore also form part of the link between the contractile ring and the plasma membrane (Jones et al., 2019). In addition, many other proteins have been found to localize to the contractile ring, such as phosphatidylinositol 4,5-bisphosphate (PIP2), which recruits and activates a variety of contractile ring-related proteins, the ESCRT (Endosomal sorting complex required for transport), which is involved in midbody abscission, and Spartin (Chatterjee et al., 2022; Hall et al., 2024; Lens and Medema, 2019; Santos et al., 2023). These proteins may be involved in membrane binding, cytoskeletal organization, or Rho regulation, perhaps because the importance of cytokinesis requires multiple redundant mechanisms to ensure contractile ring assembly and closure (Figure 4).

However, the exact physical mechanism by which the actomyosin ring closes remains a subject of intense debate. The classical “purse-string” model envisions the ring as a pre-assembled, sliding-filament structure analogous to a muscle sarcomere. Recent quantitative live-cell imaging presents compelling evidence against a strict purse-string mechanism. Instead, the field is increasingly shifting toward a “cortical flow” model, where continuous, large-scale flows of cortical actin and NMII are swept into the equatorial region, constantly remodeling the ring as it constricts (Reymann et al., 2016). We suggest that the ring should not be viewed as a static architectural entity, but rather as a highly dissipative, steady-state structure maintained by the continuous turnover and flow of NMII networks. This paradigm shift fundamentally alters our interpretation of cytokinesis from a purely localized contraction to a global whole-cell biomechanical event.

## 5 Non-muscle myosin II structural and functional abnormalities can lead to a variety of diseases

The previous three sections have discussed the structure of myosin and its essential roles in cell division, demonstrating that structural or functional abnormalities in myosin can have severe consequences. Such abnormalities manifest in two ways: first, mutations in myosin itself can directly cause various diseases; second, myosin dysfunction at the cellular level leads to failed cell division and the generation of aneuploidy, which is closely linked to tumorigenesis.

At present, studies on severe diseases caused by myosin gene mutations are mainly carried out in mouse models. Studies in mouse models have shown that disruption of the genes encoding these myosin heavy chains, MYH9, MYH10, and MYH14, respectively, results in distinct phenotypes, suggesting that NMIIA, NMIIB, and NMIIC may have distinct functions during development. NMHCIIA knockout mice fail to form polarized visceral endoderm and exhibit defects in cell-cell adhesion, which results in embryonic death before organ formation (Rodeghiero et al., 2018). Embryos that mutated or knocked out NMHCIIB survived for 14.5 days (E14.5) and were accompanied by cardiac and brain defects (Lacroix and Maddox, 2012). It has been found that there are nearly 40 mutations in the NMHCIIA head and rod domains that can cause MYH9-related diseases, and all patients with these diseases are heterozygous for MYH9 mutations (Le Clainche et al., 2001). The most common problems in patients with MYH9-related disorders are related to dysfunction of platelets, which play an important role in blood clotting and clot retraction, leading to Macrothrombocytopenia. In addition, platelets derived from patients with NMIIA mutations exhibit abnormal cytoskeletal composition and fail to form or reorganize their cytoskeletal networks upon stimulation with thrombin receptor-activating peptide (Lacroix and Maddox, 2012). To date, mutations in MYH10 (encoding NMHCIIB) have not been reported to cause disease in humans. However, pioneering work from the Adelstein laboratory demonstrated that mice homozygous for the R709C mutation in NMHCIIB (a homolog of the R702C mutation in human NMHCIIA) die at E14.5 and exhibit major morphogenetic defects (Takeda et al., 2003). Furthermore, even low wild-type NMHC II-B levels (∼6%) prevented these abnormalities, highlighting that NMHC II-B insufficiency drives cardiomyocyte cytokinesis defects. These include failure to close the ventral body wall, cleft palate, cardiac ectopia, giant omphalocele, and cardiac developmental defects (Otterpohl et al., 2020; Tullio et al., 1997). These abnormalities are reminiscent of a rare human syndrome, Pentalogy of Cantrell (Kariya et al., 2013). The MYH14 gene encoding NMHCIIC has five mutation sites that can cause deafness, and these mutations are located in the head and tail domains of myosin (Hiramatsu et al., 2021; Ji et al., 2011; Lin et al., 2024). Little is currently known about the function of NMIIC *in vivo*, and NMIIC knockout mice do not exhibit significant abnormalities (Lacroix and Maddox, 2012).

**FIGURE 5:**
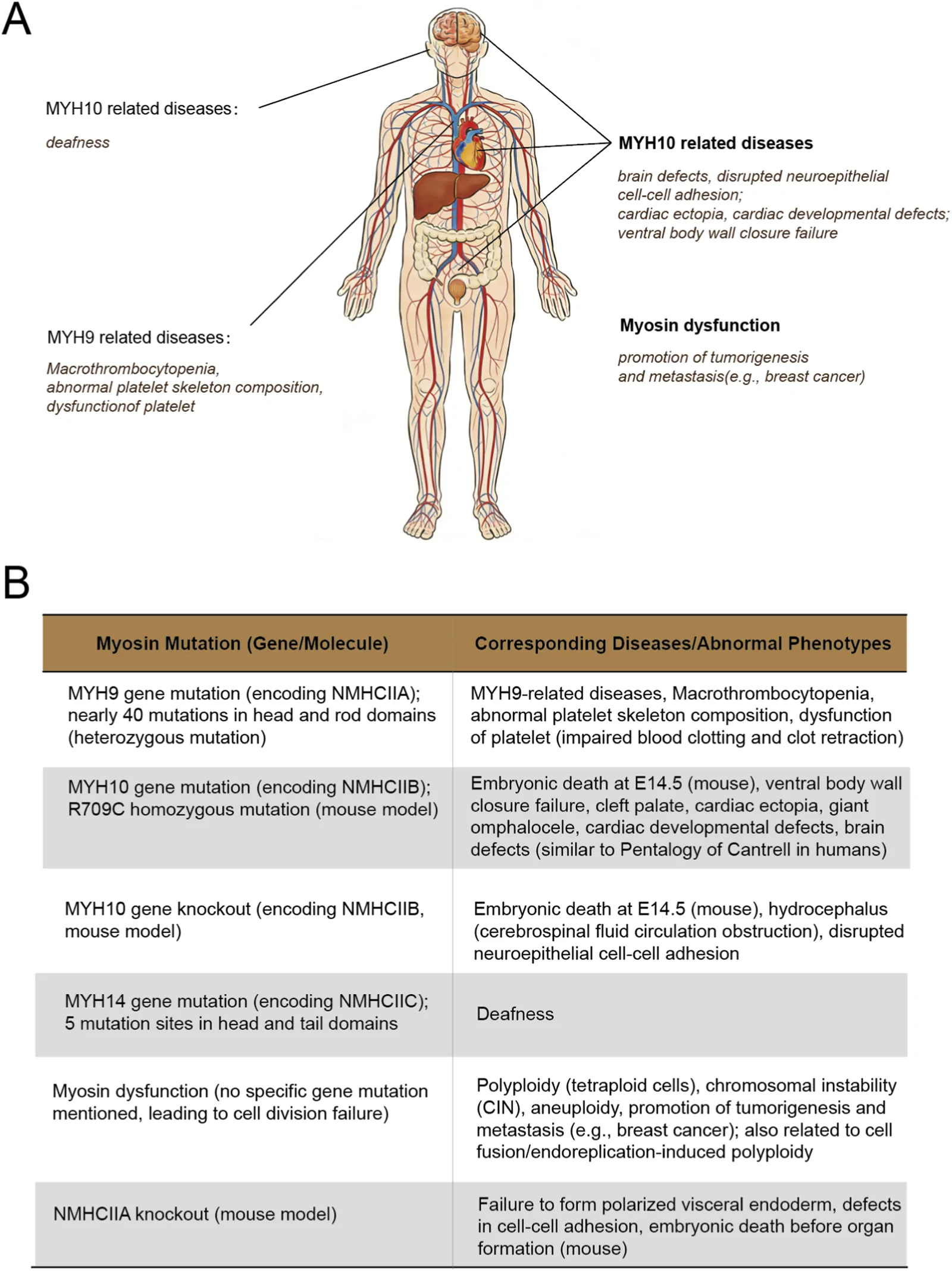
Diseases associated with mutations or abnormalities of different Myosin proteins. **(A)** Human body schematic diagram indicating the body parts affected by different myosin gene mutations. **(B)** Table listing different myosin gene mutations and their corresponding diseases.

Studies have found that the occurrence of CIN precedes the expansion and metastatic spread of several cancer subclones, and there are chromosomal aberrations in cells between almost all subclonal regions (Kim et al., 2015). Single-cell analysis of the two pairs of primary and metastatic breast cancers revealed persistent CIN in the primary breast cancer, which was dominated by aneuploid clones with a high metastatic propensity. Multi-region sequencing revealed that the majority of metastatic copy number and structural variants were derived from primary tumor subclones, and there was evidence of persistent CIN during tumor metastasis (Sansregret and Swanton, 2017). Therefore, CIN caused by cell division failure is also an important factor that promotes tumorigenesis and metastasis (Figure 5).

Given the profound implications of NMII dysfunction in driving aneuploidy and facilitating tumor metastasis, pharmacological targeting of NMII presents a promising therapeutic avenue. While early non-specific inhibitors like blebbistatin were limited by toxicity, next-generation small molecules are showing clinical potential. Recently, MT-125 has been developed as a potent dual inhibitor of NMIIA and NMIIB (Kenchappa et al., 2025). Experimental evidence demonstrates that MT-125 effectively disrupts actomyosin contractility and significantly prolongs survival in glioblastoma preclinical models, highlighting the translational value of targeting the myosin machinery in highly invasive cancers.

## 6 Discussion and prospect

Non-muscle myosin II (NMII) binds to actin to form actomyosin functional units that generate mechanical forces driving cell division, migration, and adhesion. NMII also serves as the terminal executive protein of many intracellular signaling pathways (Schiffhauer et al., 2019). The activity of NMII is tightly regulated at multiple levels, including myosin folding, filament assembly and disassembly, actin binding, and ATPase activity (Ramanathan et al., 2015). Importantly, it is an oversimplification to view NMII solely as a motor driving classical contractility. NMII acts as a multifaceted biophysical regulator: it actively modulates cellular viscoelasticity, promotes large-scale cortical flow, and drives the fluidization of actin filament networks to allow for rapid cellular remodeling. Furthermore, NMII is a central node in mechanoresponsiveness, dynamically adjusting its assembly in response to external mechanical cues. Emerging evidence even links NMII-mediated cytoskeletal dynamics to the regulation of nuclear mechanics, thereby influencing downstream gene expression and cellular metabolism. Recognizing these diverse functions is crucial for fully understanding NMII’s holistic impact on cell division (Halder et al., 2021; Huang et al., 2024; Kage et al., 2022). Moreover, NMII acts as a key effector in regulatory feedback loops that modulate the activation of upstream signaling pathways, such as those governing adhesion dynamics and actin conformational changes (Vicente-Manzanares et al., 2009). Animal models featuring isoform-specific targeted deletions have been instrumental in deciphering the distinct physiological roles of NMII isoforms and accurately modeling their associated human pathologies. However, a critical challenge in current NMII research is decoupling the functional redundancy versus specificity among its isoforms. While *in vitro* biophysical assays highlight distinct kinetic profiles for NMIIA and NMIIB, *in vivo* genetic knockout models frequently exhibit compensatory mechanisms, wherein the loss of one isoform is partially rescued by the upregulation of another. Furthermore, the historical reliance on classical pharmacological tools like blebbistatin, which possesses known phototoxicity and off-target effects, complicates the accurate interpretation of NMII mechanobiology in live cells. Future studies must use high-resolution optogenetic tools and isoform-specific degraders to precisely dissect their individual physiological contributions. Recent studies have shown that mechanical forces may reshape the tumor cell microenvironment through myosin, thereby influencing tumor development and metastasis (Feroz et al., 2024; Martinez et al., 2021; Qin et al., 2023). however, the precise mechanisms by which myosin responds to mechanical forces and remodels the tumor microenvironment remain to be elucidated. In addition, myosin serves as a central integrator of external forces during cell and tissue differentiation and cell migration. It may guide cell proliferation and differentiation by modulating microenvironmental rigidity and its own expression and activation during tissue regeneration and stem cell transplantation (Liu et al., 2020). Therefore, modulation of myosin and its associated regulatory proteins may represent a potentially valuable new approach in regenerative medicine.

Moving forward, several major open questions must be addressed to fully integrate our understanding of NMII. First, how do cells mechanically sense the completion of cytokinesis? The mechanosensory feedback loops that signal NMII disassembly upon abscission remain poorly defined. Second, while the biochemical signaling pathways (e.g., RhoA-ROCK) activating NMII are well mapped, the mechanisms by which NMII directly transduces mechanical force back into biochemical signals (mechanotransduction) within the nucleus to alter gene expression require deep exploration. Finally, understanding how aneuploid cancer cells exploit the structural plasticity of NMII to survive division failures and navigate confined microenvironments will be paramount. Answering these questions will require moving beyond static imaging toward advanced, spatiotemporally precise optogenetic and force-sensing technologies.
